# Impact of genetic variations in the WNT family members and *RUNX2* on dental and skeletal maturation: a cross-sectional study

**DOI:** 10.1186/s13005-023-00372-3

**Published:** 2023-07-03

**Authors:** Caio Luiz Bitencourt Reis, Mirian Aiko Nakane Matsumoto, Flares Baratto-Filho, Rafaela Scariot, Maria Bernadete Sasso Stuani, Fábio Lourenço Romano, Ricardo Della Coletta, Daniela Silva Barroso de Oliveira, Peter Proff, Christian Kirschneck, Erika Calvano Küchler

**Affiliations:** 1 Department of Pediatric Dentistry, School of Dentistry of Ribeirão Preto, University of São Paulo, São Paulo, Brazil; 2 School of Dentistry, Tuiuti University of Paraná, Paraná, Brazil; 3 School of Dentistry, Univille University, Joinville, Brazil; 4 Department of Stomatology, Federal University of Paraná, Paraná, Brazil; 5 Department of Oral Diagnosis, School of Dentistry of Piracicaba, University of Campinas (UNICAMP), Campinas, SP Brazil; 6 Department of Clinic and Surgery, School of Dentistry, Federal University of Alfenas, Alfenas, Minas Gerais Brazil; 7 Department of Orthodontics, University of Regensburg, Franz-Josef-Strauss-Allee 11, 93053 Regensburg, Germany

**Keywords:** Craniofacial growthm dental development, Skeletal maturation, Genetic variation, WNT, RUNX2

## Abstract

**Background:**

This study evaluated if genetic variations in the *WNT* family members and *RUNX2* are associated with craniofacial maturation, investigating dental and skeletal maturity in children and teenagers.

**Methods:**

Radiographs from pre-orthodontic treatment of Brazilian patients (7 to 17 years-old) were used to assess dental (panoramic radiographs) and skeletal maturity (cephalometric radiographs). The chronological age (CA) was calculated based on the date of birth and the time the radiographs were performed. For the dental maturity analysis, the Demirjian (1973) method was used and a delta [dental age - chronological age (DA-CA)] was calculated. For the skeletal maturity analysis, the Baccetti et al. (2005) method was used and the patients were classified as “delayed skeletal maturation”, “advanced skeletal maturation” or “normal skeletal maturation”. DNA isolated from buccal cells was used for genotyping of two genetic variations in *WNT* family genes: rs708111 (G > A) in *WNT3A* and rs1533767 (G > A) in *WNT11*; and two genetic variations in *RUNX2*: rs1200425 (G > A) and rs59983488 (G > T). A statistical analysis was performed and values of p < 0.05 indicated a significant difference.

**Results:**

There were no associations between dental maturity and genotypes (p > 0.05). In the skeletal maturity analysis, the allele A in the rs708111 (*WNT3A*) was statistically more frequent in patients with delayed skeletal maturation (Prevalence Ratio = 1.6; 95% Confidence Interval = 1.00 to 2.54; p-value = 0.042).

**Conclusions:**

The rs708111 in the *WNT3A* gene impacts on skeletal maturation.

## Background

The success of orthodontic treatment in children and teenagers depends on an accurate evaluation of a patient’s growth stages, once the treatment of skeletal malocclusions is influenced by growth [1, 2]. Craniofacial growth and development in humans are controlled by complex processes with several and constant interactions among different molecular factors [3]. These events are genetically determined from the individuals’ conception to their maturation [4, 5].

Biological indicators of growth are useful biomarkers to evaluate the growth stages of children and teenagers and to diagnose changes in growth pattern [6]. The dental maturation analysis rates the degree of maturation of teeth using image examinations [7–9]. Skeletal maturation analysis by cephalometric radiograph has received growing interest as a biological indicator of bone maturation in the past years. The morphological changes of cervical vertebrae during growth has been used as a biological indicator for assessing skeletal maturation in orthodontic patients [10, 11].

Wnt signaling pathway orchestrates an essential role in the development and homeostasis of several tissues [12]. WNT signaling members, in the canonical pathway, inhibit the degradation of β-catenin, a transcriptional activator that regulates the expression of important genes for craniofacial bone and dental development [12]. WNT3A is one of the most studied canonical members [13], that presents an involvement in both osteogenesis- and odontogenesis-related cell differentiation [14]. Non-canonical Wnt signaling is calcium-dependent with activation of WNT11 promoting bone and dental morphogenesis [13, 15, 16]. The canonical and noncanonical WNT pathways stimulate RUNX2 expression [17, 18]. RUNX2 is an important protein in dental and skeletal development [19, 20], and WNT3a and WNT11 enhance the expression and function of RUNX2, promoting osteo- and odontoblastic differentiation [21, 22].

Several factors have been associated with variations in dental and bone development, such as nutritional factors [23–25] and hormonal deficiencies [26, 27]. However, the role of genetic variations on craniofacial bone and dental development have been poorly explored [28, 29]. Genetic variations in *WNT3A*, *WNT11* and *RUNX2* have already been associated with skeletal malocclusions [28] and may also be associated with bone and dental maturity. Therefore, this study evaluated the association between craniofacial maturation and genetic variations in *WNT* family members and *RUNX2*, investigating dental and skeletal maturity in children and teenagers.

## Methods

This cross-sectional study followed the Strengthening the Reporting of Genetic Association study (STREGA) statement checklist [30]. This project was previously approved by the Human Ethics Committee of the University of ************************* (01451418.3.0000.5419). Patients whose legal guardian consented to their participation, and those who gave assent to study participation, were eligible to participate.

Two sample-size calculations were performed through G*Power Version 3.1.9.6 (Franz Faul, Universität Kiel, Germany). The difference between two independent means with the parameters of alpha = 5% and power = 80% was used for both calculations. The first calculation for skeletal maturation predicts a minimum sample 100 patients (Cohen’s D = 0.37), considering 20% of loss rate. The effect size was obtained from the data by Costacurta et al. [31]. The second calculation for dental maturation predicts a minimum of 77 patients (Cohen’s D = 0.72), considering a loss rate of 20%. The effect size was obtained from Hilgers et al. [32].

Brazilian children and teenagers 7 to 17 years-old, both genders, biologically unrelated, and undergoing orthodontic treatment were screened from 2015 to 2017. Patients with previous orthodontic and/or orthopedic treatments, previous craniofacial trauma, congenital alterations, and/or metabolic disorders were not included.

The chronological age (CA) of each individual was calculated based on the date of birth on official documents and the time the radiographs were performed.

### Dental maturity analysis

For the dental maturity analysis, panoramic radiographs from patients between 7 and 16 years-old were assessed. The Demirjian et al. [8] method was used to investigate the dental maturation and to establish dental maturity. 10% of the radiographs were assessed twice by two observers, which were trained by a senior orthodontist. Weighted Cohen’s Kappa test was performed to each evaluated tooth to test intra- and inter-observer reliability. The Kappa scores ranged from 0.82 to 1.00 for intra-observer reliability and 0.79 to 1.00 for inter-observer reliability.

If a missing tooth (dental agenesis) was found in the left side, the contralateral permanent tooth of the right side was evaluated. The child was excluded from the study if bilateral congenital agenesis was found. Dental maturity was measured subtracting the dental age (DA) from chronological age (DA - CA). Values close to or equal to 0 indicate that patient have dental maturity coincident with the chronological age. Values far from 0 indicate that the patient has a delayed (negative values) or advanced (positive values) dental maturity.

### Skeletal maturity analysis

For the skeletal maturity analysis, cephalometric radiographs from patients aged 7 to 17 years-old were assessed. The method according to Baccetti et al. [33] was used to investigate skeletal maturation. 10% of the radiographs were assessed twice by two observers, which were trained by a senior orthodontist. Weighted Cohen’s Kappa test was performed to test the intra- and inter-observer reliability, which was equal to 0.783 and 0.823, respectively.

The patients were classified as “delayed skeletal maturation”, “advanced skeletal maturation” or “normal skeletal maturation” depending on the result of Baccetti’s analysis and chronological age. The study of Schoretsaniti et al. [34] was used to establish a parameter to this classification for boys and girls separately, which is shown in Table 1.

**Table 1:** Estimated age for the boys and girls for each skeletal maturation score

Baccetti’s Scores	CS1	CS2	CS3	CS4	CS5	CS6
Estimated Age for the boys	< 10	10 to 11	11 to 12	12 to 15	15 to 17	> 17
Estimated Age for the girls	< 9	9 to 10	10 to 11	11 to 14	14 to 17	> 17

Note: This estimation was interpreted according to Schoretsaniti et al. (2021). CS means cervical stage

### DNA extraction and genotyping

Genomic DNA isolated from buccal epithelial cells was used for genotyping analysis. The saliva samples were collected from each child using saline solution, which was used to rinse [33]. Genomic DNA was then extracted as previously described [35]. The concentration and purity of the genomic DNA was determined by spectrophotometry (Nanodrop 1000; Thermo Scientific, Wilmington, DE, USA).

Two genetic variations in *WNT* family genes, rs708111 (G > A) in *WNT3A* and rs1533767 (G > A) in *WNT11*, and two genetic variations in *RUNX2*, rs1200425 (G > A) and rs59983488 (G > T), were chose. These genetic variants were selected due to their potential relevance in osteogenesis and odontogenesis-related cell differentiation [28, 36]. Genotyping was blindly performed with the Taqman™ method for real-time PCR in the StepOnePlus™ (Applied Biosystems™, Foster City, CA, USA) as previously described [35]. 10% of the sample were genotyped twice and an agreement of 100% was observed.

### Statistical analysis

Dental maturity (delta DA - CA) was evaluated as a continuous variable, while skeletal maturity was evaluated as a categorical variable. Mann-Whitney U test was used to compare dental maturity between genders, and Chi-square test was used to compare skeletal maturity between genders using IBM SPSS version 25.0 (IBM Corp. Armonk, USA). Chi-square test was also applied to calculate the Hardy-Weinberg equilibrium.

**Fig. 1:**
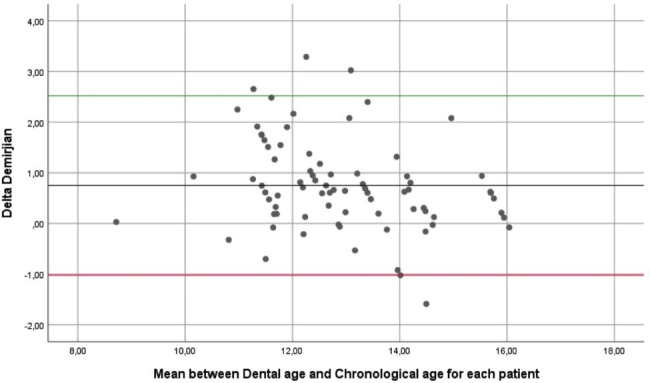
Bland-Altman Plot

To determine the agreement between CA and DA, a Bland-Altman analysis was performed. A Bland-Altman plot was generated (Fig. 1) and a linear regression was performed to evaluate proportional bias between CA and DA [37]. The linear regression indicated that a bias exists between CA and DA estimated by Demirjian (p = 0.018) that shows an overestimation of the method.

The allelic and genotypic distributions between skeletal maturation groups were performed by PLINK 1.9 software using chi-square or Fisher tests. Prevalence Ratios (PR) and 95% Confidence Intervals (95% CI) were calculated for genotype distribution among skeletal maturity groups. Mann-Whitney U test was applied for dental maturation values according to the genotypes.

Values of p < 0.05 indicated a statistically significant difference.

## Results

**Fig. 2:**
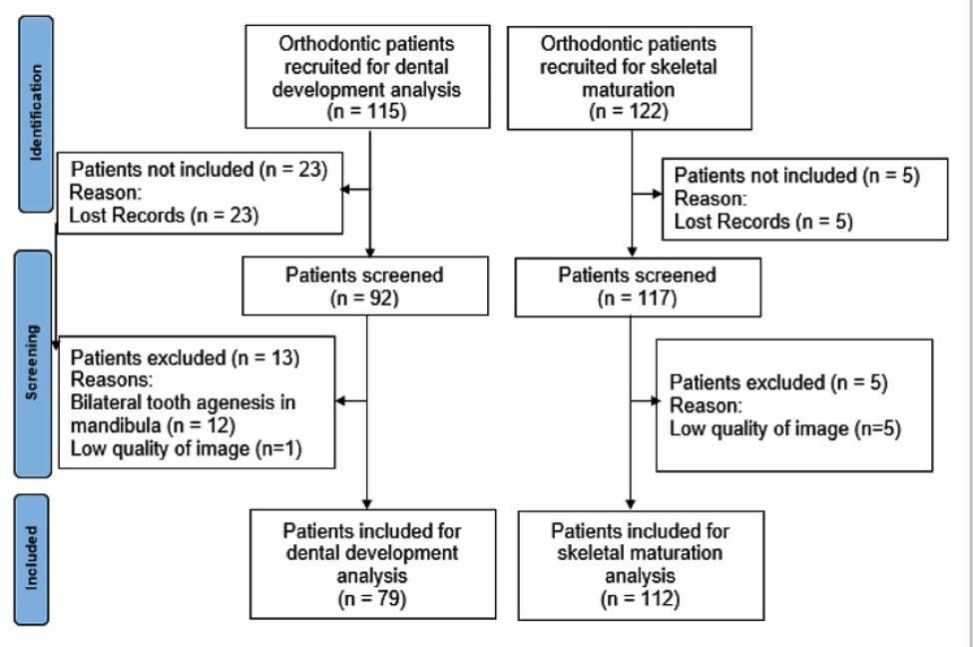
Flow diagram for both sets

The Fig. 2 shows the flow diagram for both sets. Among the 152 patients screened, 79 were included for the dental maturity set, and 101 were included for the skeletal maturity set. Table 2 shows the comparison of skeletal maturity and dental maturity between genders. There are no differences between gender in both sets (p > 0.05). The Demirjian method overestimated the age of patients in 0.75 years, on average.

**Table 2:** Characteristics of the both studied sets

Sample	Variables		Total	Male	Female	p-value	
Dental Maturity	N (%)		79 (100)	35 (44.30)	44 (55.70)		
	Chronological Age - mean (SD)		12.57 (1.68)	12.41 (1.81)	13.14 (1.58)	0.485¹	
	Delta (DA-CA) - mean (SD)		0.75 (0.90)	0.72 (0.90)	0.77 (0.91)	0.339¹	
Skeletal Maturity	N (%)		101 (100.0)	44 (43.56)	57 (56.44)		
	Chronological Age - mean (SD)		15.17 (7.39)	15.36 (7.40)	14.98 (7.42)	0.376¹	
	Skeletal maturation status distribution – n (%)	Normal	58 (57.40)	22 (50.00)	36 (63.16)	0.093²	
		Delayed	28 (27.70)	17 (38.64)	11 (19.30)		
		Advanced	15 (14.90)	5 (11.36)	10 (17.54)		

Notes: ^1^Mann-Whitney test was performed. ^2^Chi-square test was performed. * statistical significance (p < 0.05)

The studied genetic variants were within the Hardy-Weinberg equilibrium (p > 0.05).

The studied genetic variants in *WNT3A, WNT11* and *RUNX2* were not associated with dental maturity (p > 0.05). The results are presented in the Table 3.

**Table 3:** Means of delta (DA-CA) according to the genotypes for the dental maturity set

Genetic variations (Gene)	Model	Genotype	n	Median	25th Percentile	75th percentile	p-value
rs708111 (*WNT3A*)	Co-Dominant	GG	22	0.415	-0.160	0.748	Ref.
		AG	33	0.801	0.474	1.547	0.235
		AA	18	0.670	0.189	1.316	0.946
	Dominant	GG	22	0.415	-0.160	0.748	Ref.
		AG + AA	51	0.745	0.243	1.510	0.385
	Recessive	GG + AG	55	0.643	0.221	1.033	Ref.
		AA	18	0.670	0.189	1.316	0.630
rs1533767 (*WNT11*)	Co-Dominant	GG	31	0.595	0.194	0.930	Ref.
		AG	25	0.643	0.128	1.547	0.614
		AA	2	0.782	0.626	0.939	0.136
	Dominant	GG	31	0.595	0.194	0.930	Ref.
		AG + AA	27	0.643	0.128	1.547	0.882
	Recessive	GG + AG	56	0.599	0.187	0.958	Ref.
		AA	2	0.782	0.626	0.939	0.098
rs1200425 (*RUNX2*)	Co-Dominant	GG	30	0.679	0.126	1.033	Ref.
		AG	26	0.397	0.128	0.985	0.480
		AA	14	0.609	0.284	0.875	0.724
	Dominant	GG	30	0.679	0.126	1.033	Ref.
		AG + AA	40	0.573	0.156	0.957	0.499
	Recessive	GG + AG	56	0.616	0.127	1.009	Ref.
		AA	14	0.609	0.284	0.875	0.965
rs59983488 (*RUNX2*)	Co-Dominant	GG	55	0.602	0.115	1.033	Ref.
		TG	13	0.745	0.605	0.967	0.245
		TT	2	0.116	-0.121	0.352	0.321
	Dominant	GG	55	0.602	0.115	1.033	Ref.
		TG + TT	15	0.712	0.352	0.967	0.479
	Recessive	GG + TG	68	0.619	0.187	1.009	Ref.
		TT	2	0.116	-0.121	0.352	0.253

Notes: Mann-Whitney test was performed

Table 4 shows genotype and allele distribution among skeletal maturity groups. For rs708111 (*WNT3A*), the allele (A) was statistically more frequent in patients with delayed skeletal maturation than the wildtype allele (G) (PR = 1.6; 95% CI = 1.00 to 2.54; p = 0.042). There was no association between skeletal maturity and the genetic variants rs1533767 (*WNT11*), rs1200425 and rs59983488 (*RUNX2*).

**Table 4:** Genotype distribution among skeletal maturity groups

Genetic variation (Gene)	Model	Genotype	Normal		Delayed		p-value (Normal vs. Delayed)	Advanced		p-value (Normal vs. Advanced)
			n	%	n	%		n	%	
rs708111 (*WNT3A*)	Co-Dominant	GG	18	33.96	5	19.23	Reference	1	6.7	Reference
		AG	23	43.40	10	38.46	0.685	10	66.7	0.064^#^
		AA	12	22.64	11	42.31	0.066	4	26.6	0.096
	Dominant	GG	18	33.96	5	19.23	Reference	1	6.7	Reference
		AG + AA	35	66.04	21	80.77	0.175	14	93.3	0.064^#^
	Recessive	GG + AG	41	77.36	15	57.69	Reference	11	73.3	Reference
		AA	12	22.64	11	42.31	0.070	4	26.7	0.747
	Allele	G	59	55.66	20	38.46	Reference	12	40.00	Reference
		A	47	44.34	32	61.54	0.042*	18	60.00	0.131
rs1533767 (*WNT11*)	Co-Dominant	GG	25	60.98	7	38.89	Reference	6	50.0	Reference
		AG	13	31.71	11	61.11	0.059	6	50.0	0.330
		AA	3	7.32	0	0.00	†	0	0.0	†
	Dominant	GG	25	60.98	7	38.89	Reference	6	50.0	Reference
		AG + AA	16	39.02	11	61.11	0.116^#^	6	50.0	0.501
	Recessive	GG + AG	38	92.68	18	100.00	Reference	12	100.0	Reference
		AA	3	7.32	0	0.00	†	0	0.0	†
	Allele	G	63	76.82	25	69.44	Reference	18	75.00	Reference
		A	19	23.18	11	30.56	0.398	6	25.00	0.853
rs1200425 (*RUNX2*)	Co-Dominant	GG	20	37.74	7	30.43	Reference	6	42.86	Reference
		AG	24	45.28	8	34.78	0.826	7	50.00	0.785
		AA	9	16.98	8	34.78	0.266	1	7.14	0.709#
	Dominant	GG	20	37.74	7	30.43	Reference	6	42.86	Reference
		AG + AA	33	62.26	16	69.57	0.541	8	57.14	0.726#
	Recessive	GG + AG	44	83.02	15	65.22	Reference	13	92.86	Reference
		AA	9	16.98	8	34.78	0.087	1	7.14	0.358
	Allele	G	64	60.38	22	47.83	Reference	19	67.86	Reference
		A	42	39.62	24	52.17	0.151	9	32.14	0.468
rs59983488 (*RUNX2*)	Co-Dominant	GG	18	34.62	11	44.00	Reference	7	50.00	Reference
		TG	13	25.00	3	12.00	0.320	4	28.57	0.972
		TT	21	40.38	11	44.00	0.982	3	21.43	0.321
	Dominant	GG	18	34.62	11	44.00	Reference	7	50.00	Reference
		TG + TT	34	65.38	14	56.00	0.763#	7	50.00	0.292#
	Recessive	GG + TG	31	59.62	14	56.00	Reference	11	78.57	Reference
		TT	21	40.38	11	44.00	0.426#	3	21.43	0.190#
	Allele	G	49	47.12	25	50.00	Reference	18	64.29	Reference
		T	55	52.88	25	50.00	0.737	10	35.71	0.106

Notes: Normal group was used as control for comparisons. Chi-square test was performed, except p-values with ^#^, which was performed by Fisher test. * statistical significance (p < 0.05). † means that the test was not performed

## Discussion

In order to successfully treat patients, orthodontists require a comprehensive understanding of craniofacial growth and development. This knowledge allows them to identify specific growth phases and accurately estimate the remaining growth, aiding successful treatment outcomes. Therefore, the orthodontist needs to analyze the developmental status of each patient [1]. Although it is well known that genes play an important role in growth and development [38, 39], the function of key genes on dental and skeletal maturity have not been completely investigated yet. Thus, in the present study, we investigated if genetic variations in *WNT* family members and *RUNX2* impact dental and skeletal maturation.

Demirjian’s method [8] is the most widespread method to assess dental maturation / dental age, and was used to assess dental maturity in our study. However, the overestimation of the method is an important bias not only observed in this study, but also in another Brazilian sample [40], and in a systematic review with global population [41]. The overestimation may explain the proportional bias in this study between CA and DA shown through Bland-Altman analysis. The overestimation may also be due to the fact that the method being created to a French–Canadian data set. Genetic, socioeconomic and environmental variations observed in different counties may generate inconsistencies in this method, when applied in other populations [41].

The skeletal maturation assessment through morphologic alterations in cervical vertebrae has been receiving growing interest in clinical orthodontics, since it prevents an additional X-ray exposition for the patient and the pubertal spurt of facial bones coincides with the spurt of skeletal growth [1, 2]. Although the method is not considered the gold standard and the reproducibility is questionable [2], Baccetti’s method is used worldwide to predict the remaining growth of children and teenagers. In this study, we complement Baccetti’s method with the investigation of Schoretsaniti et al. [34], which observed a mean and confidence interval of chronological age for each stage of growth according to Baccetti. We observed that 42.60% of the sample were classified with a skeletal maturation alteration, in which 27.70% classified as delayed skeletal maturation.

WNT3A and WNT11 are important signalizing mediators involved in the transcription activation of many genes [42], such as *RUNX2* [21]. These proteins have already been associated with osteogenesis- and odontogenesis-related cell differentiation [14–16]. WNT3A induces dental follicle cell differentiation in cementoblastic/osteoblastic cells, acting directly in tooth and bone development in murine model [14]. WNT11 is also involved in odontoblast and osteogenic cell differentiation [15, 16]. Moreover, in vitro studies demonstrated that WNT11 and WNT3A are associated with the increase in expression and function of RUNX2 [17, 18]. When added exogenously in cultured rat primary osteoblast and mesenchymal stem cells, WNT3A and WNT11 elevate the levels of RUNX2, which promotes osteoblastic differentiation and mineralization [17, 18]. Interestingly, β-catenin, the central component of the WNT canonical pathway, was associated with odontoblastic differentiation of dental pulp cells through RUNX2 activation [22]. The association between RUNX2 and WNT noncanonical pathway on dental development is still under investigation, however, the proteins have already been separately associated with odontoblastic differentiation [19, 43]. In this way, we hypothesized that genetic variations in *WNT3A*, *WNT11* and *RUNX2* might impact dental and skeletal development and maturation time.

We investigated only one genetic variation in *WNT3A* and *WNT11* genes, which can be a limitation of this study. The variant allele (A) of the rs708111 (*WNT3A)* has already been associated with growth patterns/skeletal malocclusion [28]. In our study, this allele was associated with delayed skeletal maturation. The rs708111 (*WNT3A)* variant is located in a regulatory region [44], thus, it is reasonable to hypothesize that this variation alters the expression of WNT3A mediator and impact the skeletal development [45]. The rs1533767 in *WNT11* gene is a silent mutation [44]. This variation may change the mRNA processing impacting the exonic splicing enhancer [45]. This variant has already been associated with skeletal pattern [28], and as a protective factor for oral cancer [36]. In this study, the rs1533767 in *WNT11* was not associated with dental nor skeletal maturity. It is possible to hypothesize that other genetic variations in WNT family members affect dental maturity. Therefore, future studies should focus in the evaluation of the impact of these genes on dental maturity.

The genetic variations rs1200425 and rs59983488 in *RUNX2* were previously associated with skeletal malocclusion [28] and were selected for investigation in this study. The rs1200425 is an intronic variant that may induce aberrant mRNA splicing, and rs59983488 is an upstream variant, which is located 5’ UTR of the gene [39]. An upstream variant may affect directly the gene expression level [28]. In this study, these genetic variants were not associated with dental and skeletal maturation. Due to the impact of these variants on craniofacial development showed by previous results [28, 46], future studies should investigate their impact on dental and skeletal phenotypes in other populations.

## Conclusion

The rs708111 in *WNT3A* gene impacts the skeletal maturation. This genetic variation may predict skeletal maturation delay and impact the orthodontic treatment plan. In the future, orthodontists may be able to use genetic biomarkers to predict the growth potential of each patient.

## Data Availability

The datasets used and/or analyzed during the current study are available from the corresponding author on reasonable request.
